# A Comprehensive Approach to Exciton Delocalization and Energy Transfer

**DOI:** 10.1021/acs.jctc.2c00980

**Published:** 2022-12-23

**Authors:** D. Giavazzi, S. Saseendran, F. Di Maiolo, A. Painelli

**Affiliations:** Department of Chemistry, Life Science and Environmental Sustainability, Università di Parma, 43124 Parma, Italy

## Abstract

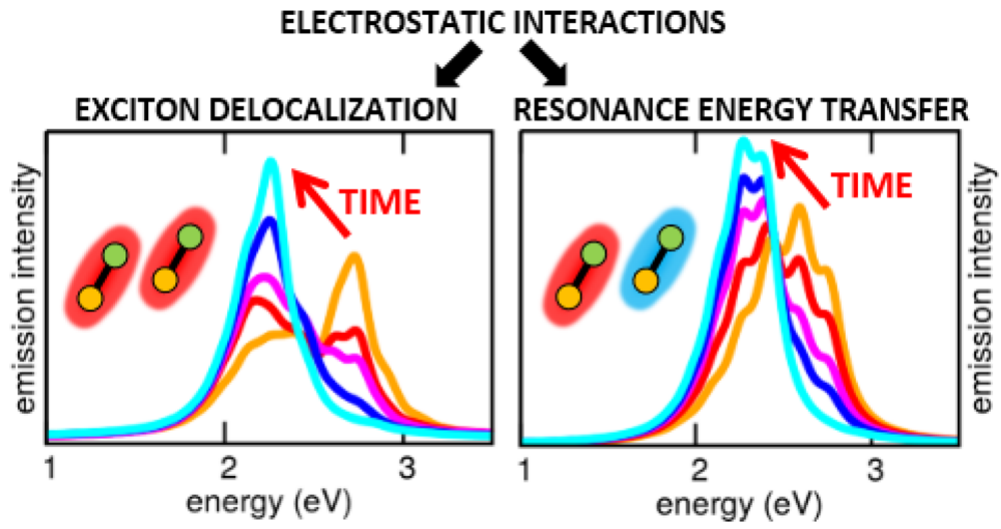

Electrostatic intermolecular interactions lie at the heart of both the Förster model for resonance energy transfer (RET) and the exciton model for energy delocalization. In the Förster theory of RET, the excitation energy incoherently flows from the energy donor to a weakly coupled energy acceptor. The exciton model describes instead the energy delocalization in aggregates of identical (or nearly so) molecules. Here, we introduce a model that brings together molecular aggregates and RET. We will consider a couple of molecules, each described in terms of two diabatic electronic states, coupled to an effective molecular vibration. Electrostatic intermolecular interactions drive energy fluxes between the molecules, that, depending on model parameters, can be described as RET or energy delocalization. At variance with the standard Förster model for RET and of the exciton model for aggregates, our approach applies both in the weak and in the strong coupling regimes and fully accounts for the quantum nature of molecular vibrations in a nonadiabatic approach. Coupling the system to a thermal bath, we follow RET and energy delocalization in real time and simulate time-resolved emission spectra.

## 1 Introduction

Resonance energy transfer (RET) and exciton delocalization play a prominent role in defining the spectral features and more generally the behavior of molecular materials.^1−7^ The two phenomena do not require delocalized electrons among involved molecules but can be driven by classical electrostatic intermolecular interactions. In this limit, very useful phenomenological models have been proposed and are widely applied to address the two phenomena, namely the Förster model for energy transfer^8,9^ and the exciton model for energy delocalization.^3,4,10^ While the two models were proposed independently, both rely on the same main approximations: (a) only states with one excited molecule are accounted for and (b) electrostatic intermolecular interactions are treated in the dipolar approximation.

The first approximation applies to systems where excitation energies are larger than interaction energies. It has been discussed quite extensively with reference to molecular aggregates,^11,12^ where it is responsible for the apparent deviations from the sum rules of the oscillator and rotational strengths. In aggregates of polar molecules,^13−16^ it leads to an offset of the excitation energies that, in some cases, may overcome the exciton splitting leading to the puzzling observation of nonfluorescent J-aggregates or of fluorescent H-aggregates. The same approximation is much less discussed in RET systems and will be addressed below.

The second approximation applies when intermolecular distances are much larger than molecular dimensions, and it is therefore poor in most cases. However, it is often adopted, as it allows for expressing intermolecular interactions in terms of the experimentally accessible transition dipole moments. Several strategies have been adopted to relax the dipolar approximation, ranging from the use of the extended dipole approximation^17^ to more refined transition density approximations.^18−20^ The most striking effects of the dipolar approximation are observed in RET, where it imposes that only optically bright states can participate in RET, a limitation that is overcome when the dipolar approximation is released.^17,21,22^

Molecular vibrations add further complexity to the picture. Their role in defining the band shape of optical spectra of molecular aggregates is well understood,^11,23−25^ as well as in allowing the weak and largely red-shifted emission of H-aggregates. In RET systems, the role of molecular vibrations was traditionally relegated to the broadening of absorption and fluorescence spectra and then favoring the Förster overlap.^26^ More recently, molecular vibrations in RET were explicitly addressed^27−35^ to account for subtle effects, including coherent oscillations, and a major role of vibrational states has been observed.^30,36^ In either molecular aggregates or RET systems, the explicit inclusion of molecular vibrations is highly nontrivial as the adiabatic approximation breaks down, and a fully quantum nonadiabatic approach to the coupled electron-vibration problem must be adopted.

In this work, we discuss a simple and reliable model that describes in a unique framework both molecular aggregates and RET. The model relaxes the two approximations of the exciton and Förster models and explicitly accounts for nonadiabatic molecular vibrations coupled to a thermal bath. Accordingly, the energy fluxes after photoexcitation are addressed solving the tricky issue of recognizing the fluorescent state and hence opening the way to calculate time-resolved emission spectra of dye aggregates and FRET systems.

The paper is organized as follows. First, in Sec. 2, we present our model for the supramolecular assembly, making a clear connection with the widely used exciton and Förster models. In Sec. 3, we discuss the dynamical model used. In Sec. 4, we present the main results of this paper accounting for both homodimers (Sec. 4.1) and heterodimers (Sec. 4.2), as well as increasingly asymmetric dimers (Sec. 4.3). Finally, Sec. 5 concludes the paper.

## 2 The Supramolecular Model

**Figure 1:**
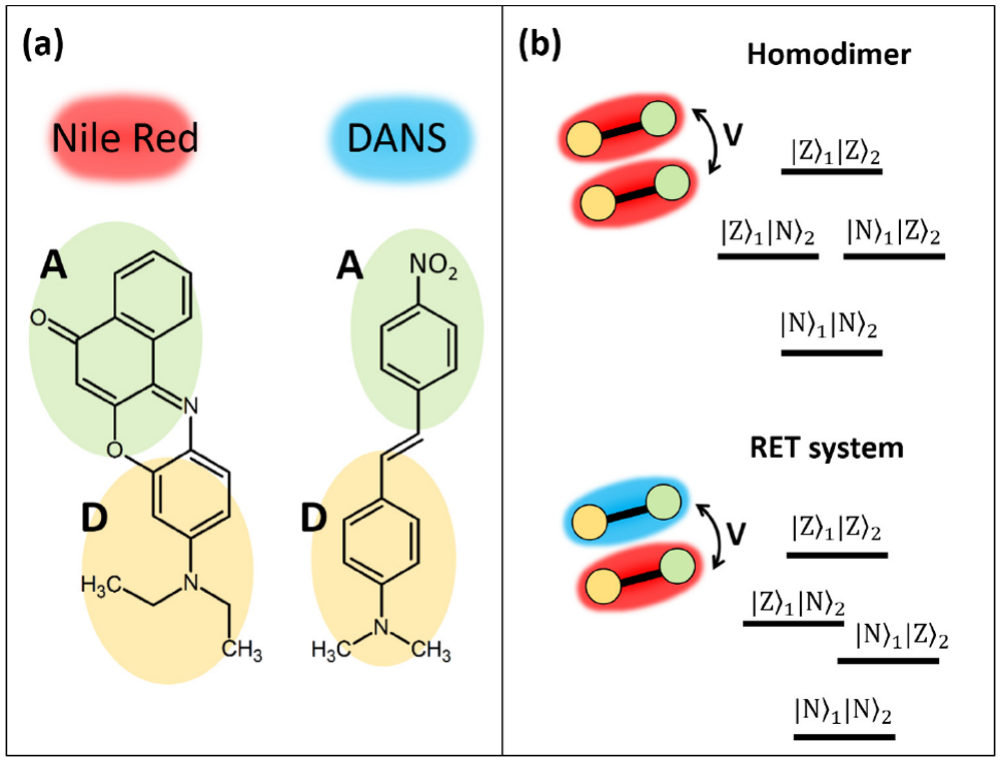
(a) Molecular structures of two representative DA dyes. (b) Sketch of the four-state electronic models used to describe two either identical (upper part) or different (lower part) interacting DA dyes.

Either in the exciton model for aggregates or in the Förster model for RET systems, each molecule is described accounting for just the ground and a single excited state. We go in the same direction, accounting for just two molecular states, that however are selected as diabatic states. Specifically, we adopt a two state model as relevant to donor–acceptor (DA) dyes, a family of molecules where an electron acceptor group and an electron donor group are joined by a π-conjugated bridge (see Figure 1a). The two diabatic basis states then correspond to the two main resonance structures, the neutral DA and zwitterionic D^+^A^–^ structures, |*N*⟩ and |*Z*⟩, respectively.^37,38^ The two states, separated by an energy gap 2*z*, are mixed by a matrix element – τ to give a ground |*G*⟩ and an excited state |*E*⟩ on each molecule. These two states correspond to the ground and excited states typically accounted for, on each molecule, in either the exciton model (that applies when the two molecules are equivalent or almost so) or in the Förster model (that applies when the two molecules are distinctively different). At variance with either the exciton model or the Förster model, however, in our case, the nature of the ground and of the excited state in each molecule (i.e., the amount of mixing between |*N*⟩ and |*Z*⟩) varies as the result of the interaction with the nearby molecule. In this view, our model accounts for the molecular polarizability, which is disregarded in either the standard exciton or Förster models.

An effective molecular vibration is introduced on each molecular site to account for the different geometries of the molecule in the two basis states. Since we will adopt a nonadiabatic approach to the coupled electronic and vibrational problem, the molecular coordinate is a quantum operator
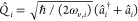
1where *â*_*i*_^†^ and *â*_*i*_ are the vibrational creation and annihilation operators, respectively, and ω_*v*,*i*_ is the frequency associated with the effective vibration on the *i*-th molecule. The strength of the electron-vibration coupling is measured by ϵ_*v*,*i*_, the vibrational relaxation energy associated with the electron transfer from |*N*⟩ to |*Z*⟩ on the *i*-th molecule. Introducing the electron-vibration coupling constant, , the molecular Hamiltonian reads
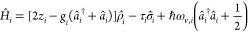
2where the index *i* = 1, 2 labels the two molecules. The electronic operators appearing in eq 2 are defined as
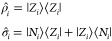
3

The molecular model in eq 2, extended to account for polar solvation, was successfully adopted to simulate linear and nonlinear optical spectra of DA dyes in solution, as well as in aggregates, leading to a reliable set of model parameters for different dyes.^37,39,40^ In Table 1, we report accepted model parameters for two well-known dyes, 9-diethylamino-5-benzo[α]phenoxazinone (Nile Red, NR) and 4-dimethylamino-4′-nitrostilbene (DANS), as shown in Figure 1a.

**Table 1:** Model Parameters for DANS^40^ and Nile Red^41^^a^

	2*z*	τ	ε_*v*_	ℏω_*v*_
DANS	2.64	0.72	0.3	0.17
Nile Red	1.76	0.95	0.33	0.14

a All the quantities are in eV.

To investigate in the same theoretical framework both energy delocalization and RET, we consider a pair of molecules that only interact via electrostatic forces. The dimer is then described in terms of four electronic basis states: |*N*_1_, *N*_2_⟩, |*N*_1_, *Z*_2_⟩, |*Z*_1_, *N*_2_⟩, and |Z_1_, *Z*_2_⟩. In line with the Mulliken approximation,^42^ we neglect the electrostatic interactions involving neutral states, so that the only surviving term, *V*, measuring the interaction between the two molecules when both are in the zwitterionic state (see Figure 1b), only affects the energy of the |*Z*_1_,*Z*_2_⟩ state that, accordingly, fully describes electrostatic intermolecular interactions in the dimer. The Hamiltonian for the molecular dimer reads
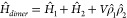
4where *Ĥ*_1(2)_ is the molecular Hamiltonian in eq 2, as relevant to the molecular site 1(2). Explicit expressions for *V* require the definition of the electrostatic model. If the dipolar approximation is considered, *V* is fully defined by the permanent dipole moments μ_0,*i*_ of the two molecules in the zwitterionic state and their relative orientation. Typical values of μ_0,*i*_ range between 10 and 30 D.^40,41,43,44^ For aligned molecules, *V* values are of the order of a few eV, for typical intermolecular distances ∼4–5 Å, similar values being obtained upon relaxing the dipolar approximation.^40^ Of course, for RET pairs, much larger intermolecular distances must be considered, leading to reduced *V* values.

The Hamiltonian for the molecular pair in eq 4 is very simple, yet it describes both energy delocalization and RET. To understand the relation between the models, following ref (16), we rewrite the dimer Hamiltonian in eq 4 on the basis of the four electronic states |*G*_1_, *G*_2_⟩, |*G*_1_, *E*_2_⟩, |*E*_1_, *G*_2_⟩, and |*E*_1_, *E*_2_⟩, with the |*G*⟩ and |*E*⟩ states defined on each molecule as
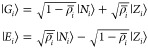
5where ρ̅_*i*_ = ⟨*G*_*i*_|ρ̂_*i*_ |*G*_*i*_⟩ is the ground state expectation value of the ionicity operator on each molecule. It is convenient to fix ρ̅_*i*_ on each molecule to its mean-field value,^16,45^ that self-consistently depends on itself (as a result of electron-vibration coupling) and on the ionicity of the nearby molecule (as a result of electrostatic intermolecular interactions)
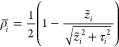
6with
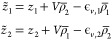
7The ground state equilibrium position for each oscillator varies with the ionicity on the relevant molecule. Accordingly, displaced bosonic creation and annihilation operators  are introduced^16^ (see Sec. S1 in the ESI).

We can now rewrite the Hamiltonian in eq 4 on the mean field basis as follows (see Sec. S2 in the ESI)
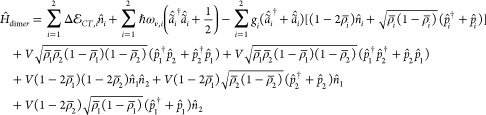
8where we introduced the Paulion operator *p̂*_*i*_ (*p̂*_*i*_^†^) that destroys (creates) an exciton on the *i*-th molecule, thus bringing it from |*E*_*i*_⟩ to |*G*_*i*_⟩ (from |*G*_*i*_⟩ to |*E*_*i*_⟩), while the exciton number operator *n̂*_*i*_ = *p̂*_*i*_^†^*p̂*_*i*_ counts the excitations on the *i*-th molecular site. The first two lines collect the on-site terms, accounting for the mean field transition energies of the dyes, , for the two displaced harmonic oscillators and (in the second row) for the linear electron–phonon coupling. All other terms, proportional to *V*, come from intermolecular electrostatic interactions. Specifically, the third line accounts for the exciton hopping term, the term that, mixing |*G*_1_, *E*_2_⟩ and |*E*_1_, *G*_2_⟩, is responsible for energy delocalization in aggregates and for energy transfer in RET systems. All terms in the subsequent lines are not accounted in the exciton or Förster model and are therefore dubbed as *ultraexcitonic*.^46^ In the fourth line, a term appears mixing the ground state |*G*_1_, *G*_2_⟩ with the state where both molecules are excited |*E*_1_, *E*_2_⟩. The fifth line describes an exciton–exciton interaction term, and the last two lines group two additional ultraexcitonic terms that mix states which differ by one exciton.

When the excitation energies are large, the ultraexcitonic terms have minor effects, and in this limit, the only relevant term accounts for the exciton migration. This term is proportional to , and in the dipolar approximation, it describes the interaction between the transition dipole moments of the two molecules. In other terms, if the dipolar approximation is adopted and ultraexcitonic terms are neglected, the model in eq 4 boils down to the standard exciton model if the two molecules are equal or to the Förster model if they are different.

The Hamiltonian in eq 8 is useful to understand the connection between the proposed model and either the exciton or the Förster models. However, working on the original diabatic basis is more expedient for the numerically exact solution of the complete problem. In the following, we therefore stick on the Hamiltonian in eq 4 and write it on the basis obtained as the direct product of the 4-dimensional electronic Hilbert space |*N*_1_, *N*_2_⟩, |*Z*_1_, *N*_2_⟩, |*N*_1_, *Z*_2_⟩, |*Z*_1_, *Z*_2_⟩ times the two Fock spaces associated with the two molecular vibrational coordinates. To make the approach numerically tractable, the basis is truncated so that the sum of the vibrational quanta associated with the two molecules does not exceed a fixed threshold *M*, high enough to ensure convergence on properties of interest (in this work *M* = 14, for a grand total of 420 basis states). Numerical diagonalization of the resulting Hamiltonian gives the numerically exact vibronic eigenstates |ϕ_*a*_⟩ of the complete Hamiltonian in eq 4 (or equivalently in eq 8), without reducing the basis set to the single exciton manifold, as usually done in the exciton model or in standard treatments of RET.

## 3 The Dynamical Model

Having a simple and reliable model for molecular dimers, we are ready to set up a dynamical approach to follow in real time RET and exciton delocalization, with the unique possibility of unambiguously identifying the fluorescent state. Indeed, the selection of the emissive state can be tricky, especially in RET systems and H aggregates. Here, we face this issue introducing a model for the *dissipative* quantum dynamics that allows us to follow the excited state dynamics. Along these lines, we will be able to address time-resolved emission spectra and to single out the Kasha’s state as the long-living excited state.

To address dissipative dynamics, the system is coupled to a thermal bath simulated as an infinite number of quantum harmonic oscillators.^47^ The coupling involves the vibrational coordinates of the system,^48^ so that the full Hamiltonian reads
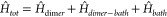
9
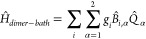
10
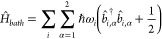
11where α runs over the two molecular units, *B̂*_*i*,α_ = (*b̂*_*i*,α_^†^+*b̂*_*i*,α_)/√2 is the *i*-th bath coordinate coupled to the α-th supramolecular vibration, with *i* ranging from one to infinity, covering all possible bath frequencies. The α-dependence acquired by the bath coordinates means that each molecular vibration *Q̂*_α_ is coupled to an independent harmonic bath.^47,49−51^ The coupling constants *g*_*i*_ (we drop the α index here for the sake of simplicity) define the strength of the system-bath coupling and enter the definition of the bath spectral density, .

The system dynamics is described by the Redfield equation^52−58^
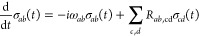
12where σ_*ab*_ = ⟨ϕ_*a*_|σ̂|ϕ_*b*_⟩ is the element of the reduced density matrix written on the vibronic eigenstates of the dimer Hamiltonian in eq 4. The first term on the right-hand side describes the unitary time evolution (i.e., the Liouville-von Neumann dynamics), with ω_*ab*_ = (*E*_*a*_–*E*_*b*_)/ℏ. The second term describes energy dissipation, the matrix elements of the Redfield tensor *R*_*ab*,*cd*_ being reported in the ESI, Sec. S3. Since solving eq 12 for large systems is computationally cumbersome, we adopt the secular approximation, only accounting for the *R*_*ab*,*cd*_ terms with |ω_*ab*_–ω_*cd*_| = 0.

The proposed model is general and applies to pairs of either identical or different molecules, as to simulate excitonic and RET pairs, respectively. However, for molecular homodimers, the high degree of degeneracy leads to divergent dynamics. To overcome this problem, two slightly different frequencies ω_*v*,1_ and ω_*v*,2_ could be assigned to the two molecules. However, this would artificially lower the symmetry of the dimeric system, heavily affecting its physics. A viable alternative to deal with homodimers recasts the system Hamiltonian in eq 4 in terms of the symmetrized vibrational coordinate operators *Q̂*_+_ and *Q̂*_–_:
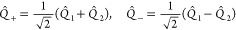
13By assigning slightly different values for ω_*v*,+_ and ω_*v*,–_, the numerical problem is addressed without breaking the symmetry. Results shown below for Nile Red dimers are obtained for ℏω_*v*,+_ = 0.15 eV and ℏω_*v*,–_ = 0.13 eV, while maintaining the vibrational relaxation energy equal to 0.33 eV (see Table 1).

## 4 Results

We take as reference systems the two dyes in Figure 1a, as representative of the large family of push–pull dyes.^41^ Relevant model parameters are listed in Table 1. Molecular Hamiltonians for different dyes will have slightly different parameters, but the differences are marginal and do not alter the main picture. In the following, we will always couple the relevant system to a quantum bath at 300 K, defined by a constant spectral density, ,^59−62^ setting γ = 5 ps^–1^ (results for a Debye spectral density are reported in Sec. S4.1 in the ESI). Eq 12 is solved using the Short-Iterative-Arnoldi (SIA) algorithm^63,64^ with a 1 fs time integration step.

### 4.1 Molecular Dimers

**Figure 2:**
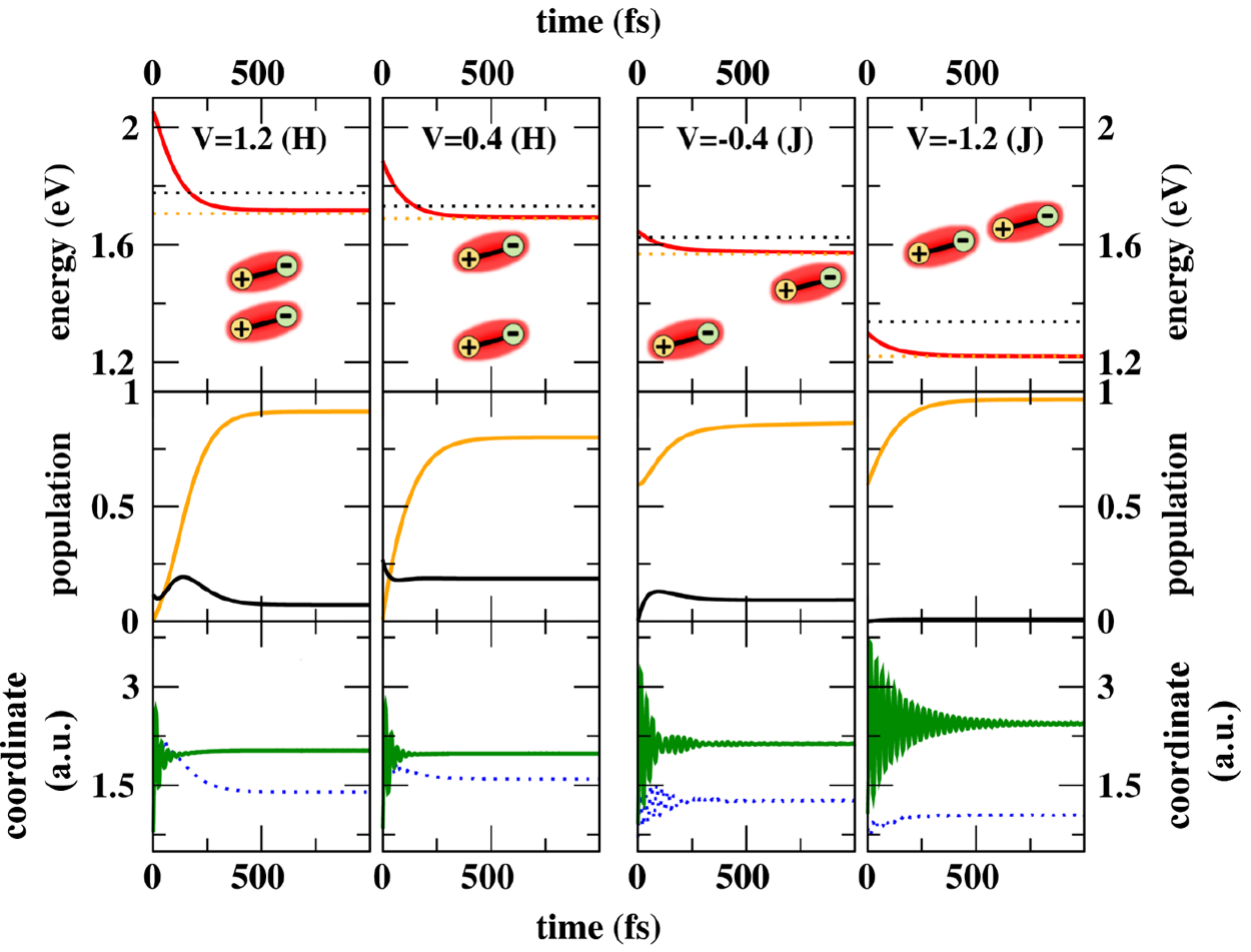
Results for dimers of NR (molecular parameters in Table 1). Each column shows results obtained for the *V* value listed in the topmost panel (eV units). Top panels: time evolution of the system energy (red). For reference, the energy of the lowest vibronic eigenstate in *S*_1_ and *S*_2_ manifolds is shown as orange and black dotted lines, respectively. The sketches in the upper panels schematically show the geometrical arrangement of the molecular permanent dipoles. Middle panels: time evolution of the populations of the lowest vibronic eigenstate in *S*_1_ and *S*_2_ manifolds (orange and black lines, respectively). Bottom panels: time evolution of ⟨*Q̂*_+_⟩ and *ΔQ*_–_ (green line and blue dotted line, respectively).

**Figure 3:**
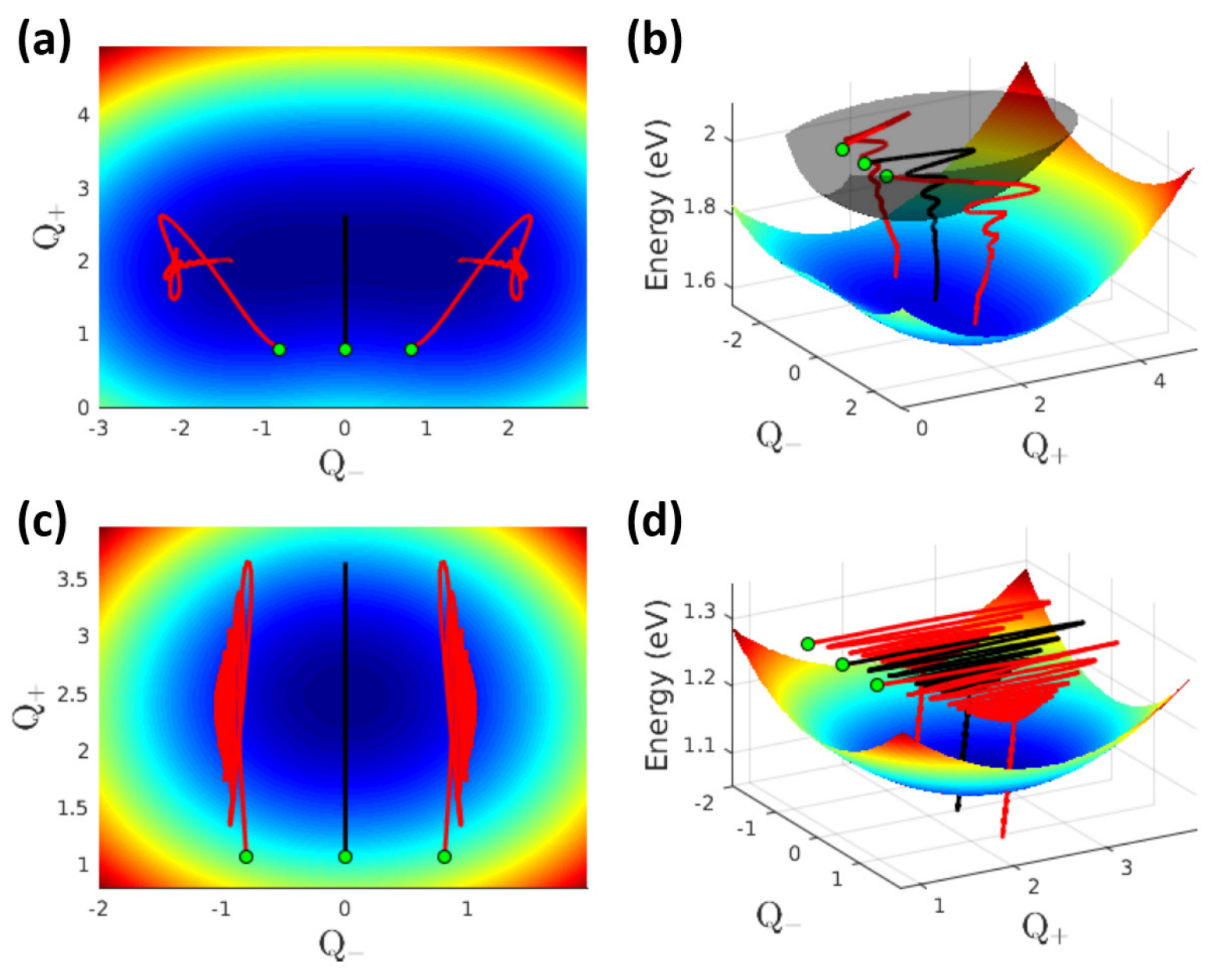
Vibrational trajectories calculated for NR dimers (same parameters as in Figure 2). Top panels refer to an H-dimer (*V* = 1.2 eV), and bottom panels refer to a J-dimer (*V* = −1.2 eV). The colored surfaces show the adiabatic potential energy surface of *S*_1_, and in panel (b), the gray surface refers to the adiabatic potential energy surface of *S*_2_. The black lines show the evolution of the system energy as a function of ⟨*Q̂*_+_⟩ and ⟨*Q̂*_–_⟩. The red lines show the evolution of the system energy as a function of ⟨*Q̂*_+_⟩ and ±*ΔQ*_–_. In all trajectories, green dots mark the starting point (*t* = 0). Note the different energy scale in panels b and d.

We start discussing the relaxation dynamics in molecular dimers, setting molecular parameters to those relevant to Nile Red (NR) in Table 1, and varying the electrostatic intermolecular interaction *V* from −1.2 to 1.2 eV. We underline that *V* measures the electrostatic interaction between the two molecules in the zwitterionic state (see Figure 1b). The interaction entering the exciton model measures instead the interaction between the transition dipole moments and is related to *V* by a simple expression *J* = *Vρ*(1−ρ) (see discussion following eq 8). For NR, ρ ∼ 0.16, so that, in the chosen *V* interval *J* ranges from ∼−0.16 to 0.16 eV, in line with typical values for molecular aggregates. Here, we only address dimers where the molecular permanent dipoles are aligned in a parallel way (noncentrosymmetric dimers), as schematically shown in the topmost panels of Figure 2. For comparison, results on dimers with antiparallel orientation of the dipoles (i.e., centrosymmetric dimers) are shown in the ESI, Figure 3S.

At time *t* = 0, the system undergoes an impulsive excitation, and the initial state for the dynamics is^47,61,62,65^
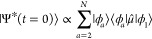
14where *a* runs over the excited eigenstates of *Ĥ*_*dimer*_ (|ϕ_1_⟩ is the ground state), and μ̂ = μ̂_1_ + μ̂_2_ is the dimer dipole moment operator for the chosen geometry. To speed up the calculation, we neglect states lying at higher energy than the most populated states reached upon coherent excitation and whose initial population is lower than 10^–6^.^47,61,62^

Figure 2 shows relevant results. As it is well-known, in J and H aggregates, the optically allowed transitions go toward the first and second electronic excited state, respectively.^7,66^ Accordingly, for negative *V* values (J-dimers), the initial excitation brings the system in the first excited electronic manifold (namely *S*_1_, see also Figure 3d, where the green dot marks the initial state of the dynamics). On the opposite, for positive *V* values (H-dimers) the most populated initial state belongs to the second electronic excited manifold, *S*_2_ (see also Figure 3b). In either case, the initially populated state is the vertical state, lying higher in energy than the lowest eigenstate in the relevant *S*_1_ or *S*_2_ manifold. A fast relaxation is always observed in the first ∼100 fs that transfers the population from the initially populated states to the lowest state of the *S*_1_ manifold, the so-called Kasha’s state. We underline that the adopted relaxation model with the two molecular coordinates coupled to two independent baths allows for the uncorrelated motion of the relevant degrees of freedom or, in other terms, allows for energy dissipation driven by either the *Q̂*_+_ or *Q̂*_–_ coordinate. This is important to allow for energy relaxation among states with different symmetries, a phenomenon that would be strictly forbidden if both molecular coordinates were coupled to a single bath (or, equivalently, if only the *Q̂*_+_ coordinate was coupled). After an initial very fast relaxation on the time scale of ∼0.1–0.5 ps, once the system reaches the Kasha’s state,^47^ the dynamics slows down dramatically with relaxation times in the nanosecond window. The different time scales of the two processes ensure that, in this case, emission occurs from a thermally equilibrated Kasha’s state, as best demonstrated by the residual thermal population of the lowest eigenstate in the *S*_2_ manifold observed at long times for *V* = 1.2, 0.4, and −0.4 eV (middle panels of Figure 2).

We now look at the expectation values of the vibrational coordinates, ⟨*Q̂*_±_⟩ = *Tr*(σ̂*Q̂*_±_), where *Tr* is the trace operation. An initial regime of coherent vibrational oscillations of ⟨*Q̂*_+_ ⟩ is observed in all dimers (bottom panels in Figure 2). Quite interestingly, the coherence lives longer in J-dimers and particularly so in J-dimers with large *V*. In these systems in fact, the dynamics in the *S*_1_ manifold is marginally perturbed by the presence of *S*_2_ states (see also Figure 3d). On the opposite, in H-dimers, the vibrational coherence is quickly lost due to the interfering relaxation toward states in the *S*_1_ manifold (see also Figure 3b).

The expectation value ⟨*Q̂*_–_ ⟩ vanishes by symmetry along the whole dynamics. Yet, its standard deviation  shows a qualitatively different behavior for J and H aggregates. This is best appreciated by data reported in Figure 3. In this figure, the color maps show the adiabatic potential energy surfaces (PESs) of the *S*_1_ state of two dimers (the gray surface in panel b shows the PES for the *S*_2_ states). In the adiabatic approximation, the vibrational kinetic energy is neglected, and the vibrational coordinates become classical variables: the PESs show the energy of the relevant state, calculated upon varying the two classical coordinates *Q*_+_ and *Q*_–_. Specifically, top panels (a and b) show results for an H dimer with *V* = 1.2 eV, and bottom panels (c and d) refer instead to a J dimer with *V* = −1.2 eV. The PES relevant to the J-dimer with *V* = −1.2 eV has a single minimum, and *ΔQ*_–_ is roughly constant as expected for a quasi-harmonic oscillator. On the opposite, the H-dimer with *V* = 1.2 eV has a largely anharmonic PES with two well developed minima. In this case, large *Q̂*_–_ fluctuations drive the system to visit the two minima, suggesting a tendency toward energy localization.

Optical absorption spectra of the dimer are calculated as the real part of the Fourier transform (FT) of the damped dipole–dipole correlation function *C*_*abs*_^*μμ*^(*t*) = ⟨μ̂(t)μ̂(0)⟩ exp[−*t*/*a*] = *Tr*{μ̂Ω̂_*abs*_(*t*)}exp[−*t*/*a*], where the absorption generating function, Ω_*abs*_(*t* = 0) = μ̂|ϕ_1_⟩⟨ϕ_1_|, evolves according to the Liouville-von Neumann (unitary) equation.^47,59,67−69^ Time-resolved fluorescence spectra are calculated allowing the system to evolve after photoexcitation and calculating the emission spectra at time *t*_1_ along the dynamics, as the FT of the damped dipole–dipole correlation function *C*_*μμ*_^*fluo*^(*t*;*t*_1_) = *Tr*{μ̂ Ω̂_*fluo*_(*t*;*t*_1_) } exp[−*t*/*a*], where the fluorescence generating function is Ω̂_*fluo*_(0;*t*_1_) = μ̂σ̂(*t*_1_) .^47,62^ To avoid spurious signals, absorption spectra are calculated using the upper triangle of the dipole moment operator, and emission spectra are calculated using the lower triangle.^70^

**Figure 4:**
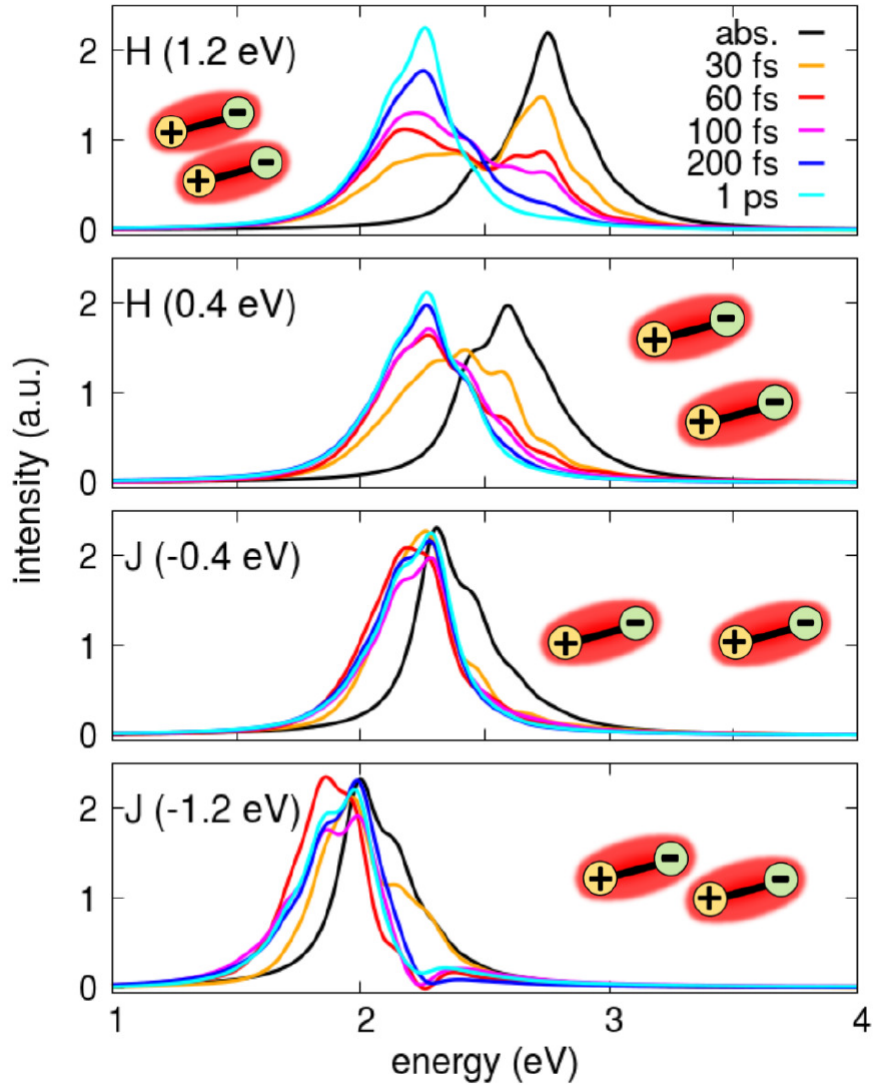
Steady state absorption (black curve) and time-resolved emission (colored curves) spectra for Nile Red dimers (same parameters as in Figure 2). All the spectra are normalized to unit area. The dipole–dipole correlation function is damped with *a* = 7 fs.

Figure 4 shows absorption and emission spectra normalized to unit area. In H-dimers, the time-resolved fluorescence shows a bimodal behavior: at a very early time, emission largely comes from the second electronic manifold *S*_2_, the same state populated upon absorption with a large transition dipole moment. As time goes on, the population is transferred to the first excited state *S*_1_ leading to a much weaker and largely red-shifted emission. Indeed we show normalized spectra, but H-dimer emission is at least 1 order of magnitude weaker than for J-aggregates. Specifically, the weak long-time emission from the dark *S*_1_ state in H-aggregate arises due to a vibronic coupling mechanism, with the emission occurring toward the ground state with a vibrational quantum in the antisymmetric *Q̂*_–_ mode.^7^ Interestingly, in the H-aggregate with *V* = 0.4 eV, a shoulder is clearly seen in the long-time emission superimposed to the 0–0 absorption band. This signal is due to the residual thermal population of the (optically allowed) lowest state in the *S*_2_ manifold (see middle panels in Figure 2).

Time evolution of the emission spectra of the J-aggregates is less prominent, and since the Kasha’s state coincides with the bright state reached upon absorption, the emission spectrum calculated at 1 ps is essentially the mirror image of the absorption spectrum, as expected. In large J aggregates, a sizable increase of the intensity of the 0–0 absorption peak vs the 0–1 peak upon increasing *V* would be expected,^23^ as a result of delocalization of the exciton on several molecules, that would imply a reduced effective electron-vibration coupling. This phenomenon is not observed in our dimeric systems, where the exciton can never spread on more than two molecules. The results are further validated by data in Figure S4 in the ESI where calculated spectra are compared with the steady state spectra as obtained in a standard sum over state approach.

### 4.2 Resonance Energy Transfer

**Figure 5:**
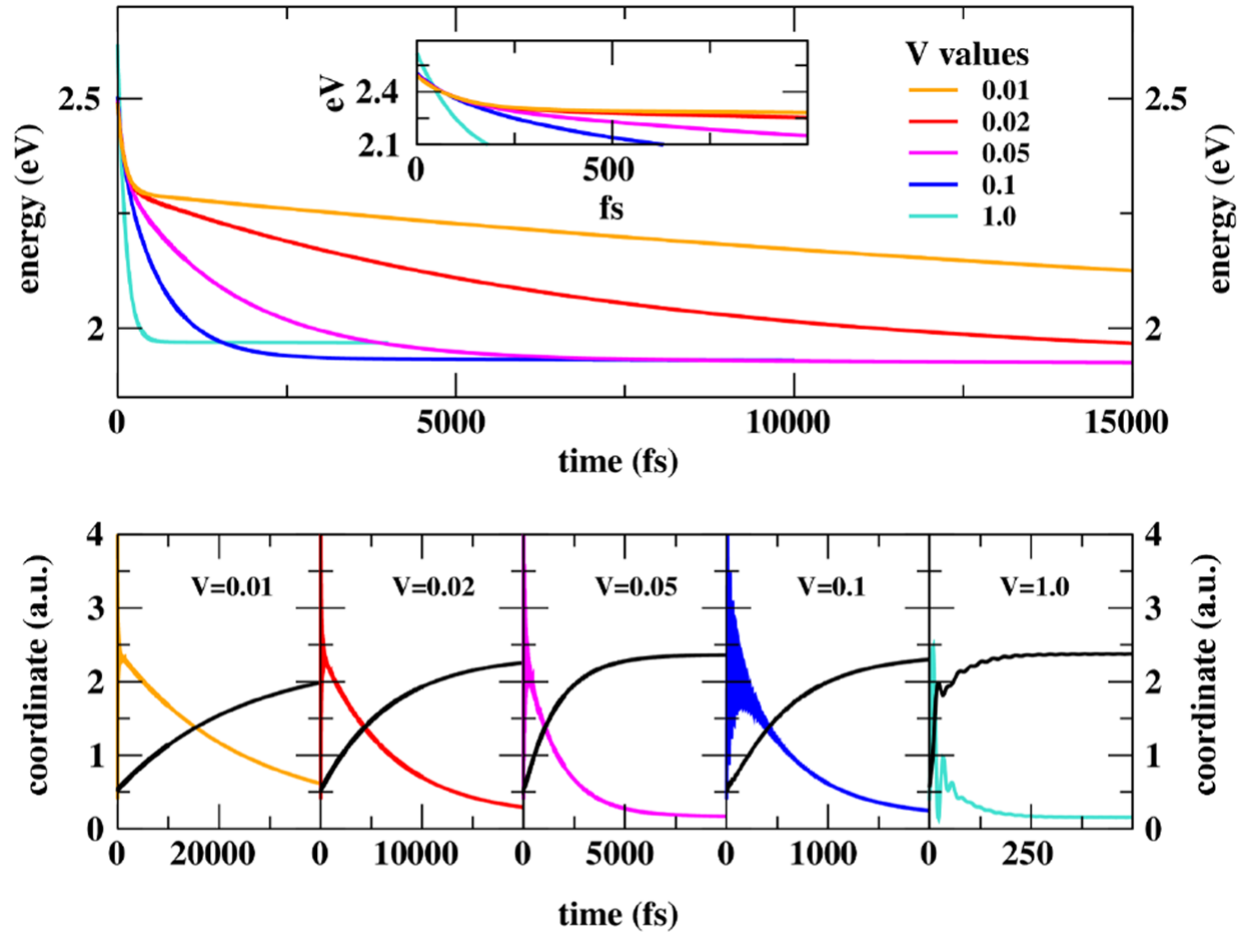
RET dynamics for a DANS-NR pair (molecular parameters in Table 1) for different values of the electrostatic intermolecular interaction *V*. Top panel: time-evolution of the system energy (the inset zooms on early time); bottom panels: time evolution of  (colored lines) and  (black lines).

The two molecules in Figure 1a form a good RET pair, with a sizable overlap between the emission spectrum of DANS, acting as the energy donor , and the absorption spectrum of NR, acting as the energy acceptor . For the sake of clarity, we will now use the  and  indices to refer to the two molecules. To address RET, we prepare the system in a state where only the energy donor  is coherently excited, inserting in eq 14 the dipole moment operator relevant to the energy donor  (results for an alternative excitation scheme are discussed in Sec. S4.3 in the ESI). Figure 5 shows results obtained for the DANS-NR RET pair with different values of the electrostatic intermolecular interaction *V*. For *V* ≤ 0.05 eV, the initial vibrational relaxation brings the system in the lowest vibronic eigenstate of the excited energy donor manifold within the first few hundreds of femtoseconds, as seen from the initial energy drop, as well as in the time evolution of : after some initial oscillations, the coordinate stabilizes at the equilibrium geometry of the excited donor. From there, the energy flows toward the energy acceptor  through an incoherent process that can take several picoseconds to reach completion (as reflected by ) depending on the interaction strength *V*. When *V* increases (*V* ≥ 0.1 eV), the system enters the strong coupling regime, with RET occurring in the same time window as the vibrational relaxation of . For *V* = 0.1 eV, RET clearly starts, while  is still coherently oscillating, meaning that energy is partially transferred also from *hot* donor states. For even larger coupling (*V* = 1 eV), RET is even faster, and the system enters a coherent transfer regime, as shown by the induced oscillations on .

The model applies in a very wide range of intermolecular interaction strenghts, providing a consistent and unified picture of RET, both in the weak (as described in the Förster model) and strong coupling regimes. By treating on the same footing vibrational relaxation, RET, and the relaxation toward the ground state, without imposing any artificial separation on the time scale of the different phenomena and accounting for both forward and backward RET, our model reliably simulates the dynamics of the system irrespective of the coupling strength.

**Figure 6:**
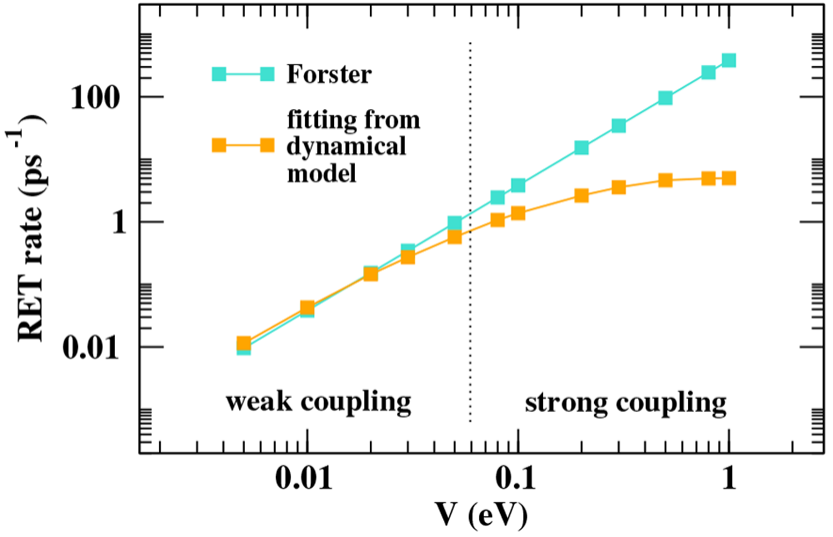
Log–log plot showing the dependence of RET rates estimated for the DANS-NR pair (molecular parameters in Table 1) over the electrostatic interaction *V*. Cyan symbols refer to estimates obtained in the Förster model; orange symbols refer to results obtained from an exponential fit of the results of the dynamical simulations (see Sec. S5 in the ESI).

In Figure 6, we compare RET rates estimated in our approach via an exponential fit of the time-dependent  state population (see Sec. S5 in the ESI) with those obtained in the Förster approximation. In the Förster model, RET occurs from the relaxed excited donor with a rate constant
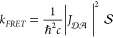
15where  is the spectral overlap between the fluorescence spectrum of the isolated donor () and the absorption spectrum of the isolated acceptor () (both shown against the wavenumber in cm^–1^ and normalized to unit area), while  is the electrostatic intermolecular interaction between the states involved in the RET process. The transition dipole moments for the isolated energy donor and acceptor are proportional to  and , respectively, so that, as discussed in Sec. 2, . Results in Figure 6 are obtained by setting  cm. In the weak coupling regime (up to *V* ∼ 5–50 meV), the dynamical model agrees well with Förster prediction. Indeed, in this regime, the RET process has characteristic times of ∼1–10 ps, at least an order of magnitude slower than typical vibrational relaxation rates. On the other hand, in the strong coupling regime, the time scale separation between vibrational relaxation and RET, as imposed in the Förster model, breaks down, and sizable deviation from Förster rates is observed.

**Figure 7:**
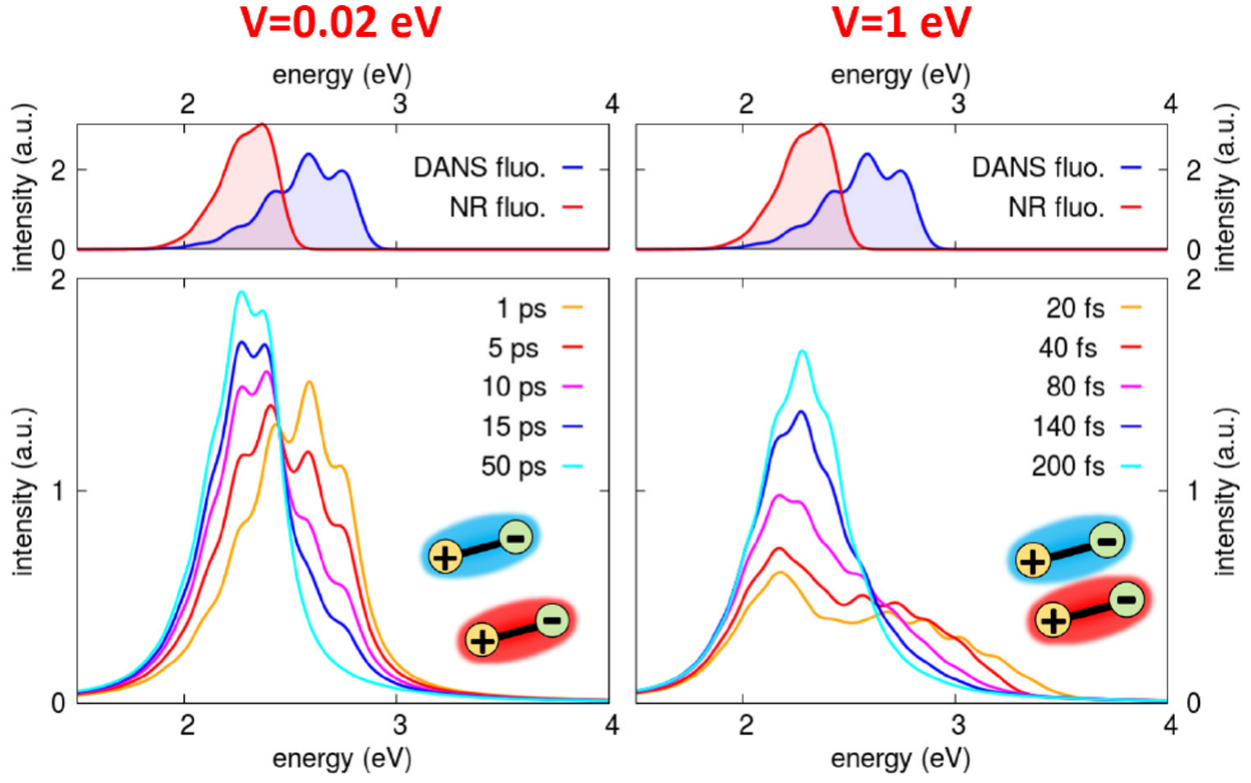
Time resolved emission spectra calculated for the DANS-NR pair (molecular parameters in Table 1, , ) in the weak (*V* = 0.02 eV) and strong coupling regime (*V* = 1 eV), left and right panels, respectively. For reference, the topmost panels show in either case the steady-state fluorescence spectra calculated for the isolated DANS and NR dyes. All the spectra are normalized to unit area.

Time-resolved fluorescence spectra offer a way to experimentally follow the RET process in time. Figure 7 shows time-resolved emission spectra calculated for the DANS-NR pair, using the same machinery described in Sec. 4.1. To facilitate the analysis, top panels of Figure 7 show steady state emission spectra of the isolated DANS and NR dyes. For weak coupling (*V* = 0.02 eV), the spectra smoothly evolve from the  spectrum to the  spectrum. Indeed, after ∼50 ps, only the  spectrum survives. For strong coupling (*V* = 1 eV), the situation is very different. The whole process occurs faster (∼200 fs), and energy is transferred to the acceptor also from the *hot* states of the donor. More to the point, the spectra cannot be considered as the simple sum of  and  spectra: in the strong coupling regime, the interaction between the two dyes heavily affects their properties. The spectra are therefore strongly perturbed, and even after the RET completion, the spectrum does not match the steady state spectrum of .

**Figure 8:**
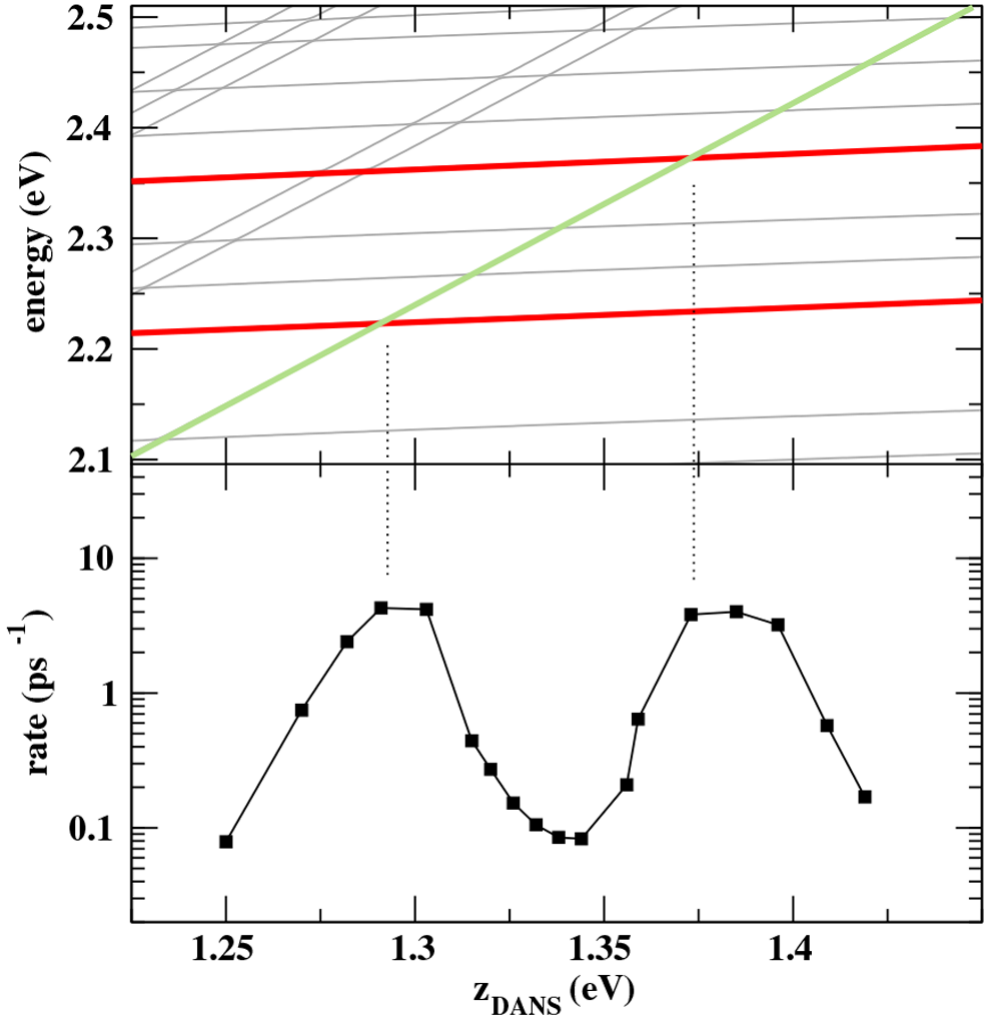
Vibronic energies (top panel) and the RET rate (bottom panel) as a function of  for the DANS-NR pair with *V* = 0.03 eV. Results are obtained with the molecular parameters in Table 1 but varying the . In the top panel, thicker curves highlight the energies of the most important states involved in the dynamics, namely Kasha’s state for the excited energy donor  (green) and the excited energy acceptor  with two and three vibrational excitations (red).

While the Förster model leads to the correct *V*-dependence of the RET rates at least in the weak coupling regimes, it does not allow to appreciate subtle phenomena related to the details of the vibronic spectrum. In the bottom panel of Figure 8, we show the variation of RET rates calculated for a system where the  parameter is varied (simulating, e.g., the effect of a local electric field), as to affect the alignment of vibronic states. We see a nonmonotonous behavior, with 2 orders of magnitude variations of the rates. To understand the physical origin of this phenomenon, in the top panel of the same figure, we show the -dependence of relevant vibronic energies. RET rates are particularly large when the energy of the long-lived Kasha’s state of  (green line), the states from where RET occurs, crosses states in the manifold relevant to the excited  with either two or three vibrational excitations (red lines), the states that mainly receive the energy. A very clear energy resonance effect is observed affecting the RET rates and highlighting the prominent role of vibronic state alignment, fully in line with recent experimental results.^30,36^

### 4.3 The RET-Exciton Crossover

Having a model that naturally encompasses energy delocalization in homodimers and energy transfer in heterodimers, we can exploit it to address the crossover regime. A first observation is that in aggregates a paramount role is played by the sign of the intermolecular interaction, *V*, that distinguishes J and H aggregates. On the opposite, the sign of *V* does not enter the Förster rate equation. This qualitative difference is easily understood: when energy delocalizes between two equivalent molecules, the sign of the interaction defines the relative energy of the in-phase and out-of-phase combinations of the two states where the excitation resides on either molecule. When the molecules are not equivalent, instead, the order of the excited states is governed by the energy difference between the two local excitations, the contribution from intermolecular interactions being marginal in most cases.

**Figure 9:**
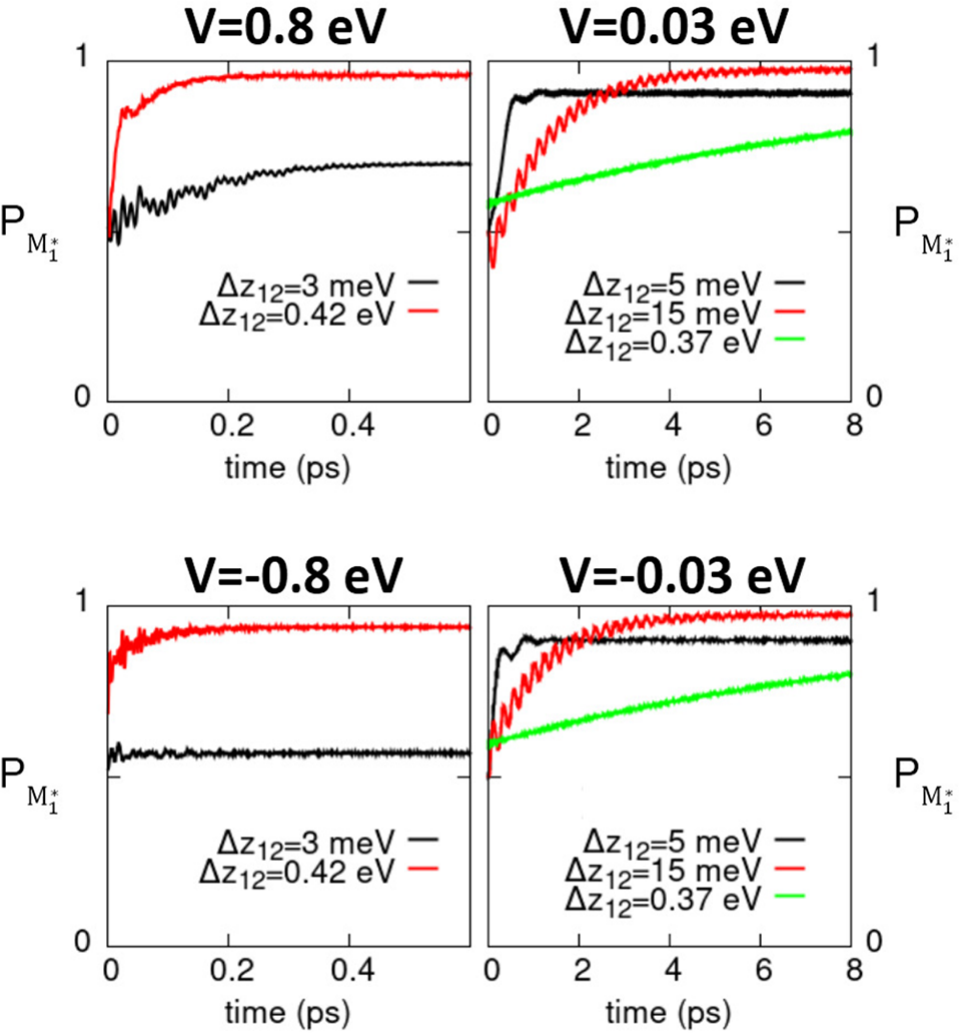
Results for NR asymmetric dimers (molecular parameters in Table 1, with the *z* value of one molecule increased by an amount *Δz*_12_). Time evolution of the global population of one of the molecules of the dimer (*P*_*M*_1_^*^_) for different *V* values and *Δz*_12_.

To investigate the crossover regime, we consider increasingly asymmetric dimers, adopting NR parameters (see Table 1) but assigning a different *z* value to one of the two molecules. The asymmetry is then defined by *Δz*_12_ = *z*_2_ – *z*_1_ (where we keep *z*_1_ fixed, while increasing *z*_2_). Figure 9 shows the time-evolution of the global population of states with the excitation residing on the first molecule *P*_*M*_1_^*^_ (see Sec. S6 in the ESI for the explicit definition) calculated for asymmetric Nile Red dimers with different *Δz*_12_ values and with positive and negative interactions and different values of *V*.

When a large *V* value is considered (|*V*| = 0.8 eV), its sign makes a difference only for small values of the asymmetry parameter, where basically the system behaves as an aggregate, with almost perfect delocalization *P*_*M*_1_^*^_ ∼ 0.5. In this case, the delocalization is slightly favored for negative *V*. For larger asymmetry, the system behaves as a RET pair, with the excitation localizing on the molecule with the lowest excitation energy, acting as the energy acceptor. The sign of *V* in this case only affects the very early time dynamics, when the relative phase of states with the excitation on either sites is relevant. For smaller *V*, the exciton delocalization regime is not regained, except for vanishing asymmetry.

## 5 Conclusions

Electrostatic interactions drive energy fluxes in assemblies of either nonequivalent or equivalent molecules. Well-established approaches to describe energy fluxes are available and proved successful, namely the Förster model for RET and the exciton model for energy delocalization. However, both models only rely on a limited basis, that only comprises states with a single excitation, and then only apply in the weak intermolecular coupling regime. The interplay between energy fluxes and molecular vibrations leads to some interesting but complex physics that requires a fully quantum nonadiabatic approach to the coupled electron-vibration problem. Against this background, the simple model in eq 4 unifies RET between different molecules and energy delocalization among identical molecular units, explicitly accounting for molecular vibrations. If the dipolar approximation is used and ultraexcitonic terms are neglected, the proposed model maps either into the standard exciton model (for equivalent molecules) or into the Förster model (for different molecules). Explicitly accounting for energy dissipation, by coupling the supramolecular system to an external bath, allows for following the time-evolution of the system as needed to calculate time-dependent properties and spectra.

In Sec. 4.1, the proposed model is applied to both J and H molecular dimers. Particularly interesting results are obtained for H aggregates where the lowest excited state is a dark state that, despite being optically inactive, is populated as a result of the system relaxation. This demonstrates that a reliable model for energy dissipation easily allows the system to transfer population between states with different symmetry. At the same time, however, the overall symmetry of the system is preserved, as demonstrated e.g. by the exact vanishing, along the whole relaxation trajectory, of the expectation value of the antisymmetric vibrational coordinate, ⟨*Q̂*_–_⟩. The subtle interplay between electronic and vibrational degrees of freedom is responsible in H aggregates for a fast decoherence of vibrational motion.

Another important result is obtained again for H aggregates. In particular, the PES relevant to the lowest excited state shows a double minimum (cf. Figure 3 panels a,b), pointing to an intrinsic instability of the dimer toward symmetry breaking (i.e., excitation energy localization). Of course, localization is only possible in the presence of the coupling to a slow coordinate (e.g., a polar solvent). The proposed model confirms this result: the relaxation dynamics of the dimer occurs along the ridge that separates the two minima, thus excluding any localization. However, large fluctuations of the system are observed, showing how the system equally visits the regions of the two minima. This dynamical effect (similar to the well-known dynamical Jahn–Teller effect^65^) requires additional studies of optical spectra of these systems, to single out experimental signatures of the phenomenon.

In Sec. 4.2, the same model is adopted to describe a RET pair. The model applies in the weak-coupling regime, where, in line with the Förster model, RET occurs from the relaxed  state, but it also describes the strong coupling regime, where the transfer is effective well before the complete  relaxation (cf. Figure 5). In this regime, the RET rates obtained from our model differ from those obtained with the Förster theory (cf. Figure 6).

Finally, in Sec. 4.3, the model naturally lends itself to address increasingly asymmetric dimers. In particular, in the strong coupling regime and for small asymmetries *Δz*_12_, a negative *V* value favors energy delocalization, thus making the dimer behave like an aggregate. Further increasing the asymmetry *Δz*_12_, the dimer becomes a RET pair (cf. Figure 9).

Further steps are planned, the most challenging and interesting being the inclusion of the effects due to a polar environment (e.g., a polar solvent) in a fully quantum and dynamical way. Indeed, the photophysics of supramolecular assemblies can be strongly affected by the interactions with the surroundings.^71,72^ Moreover, having a fully dynamical picture of symmetry breaking induced by solvation effects in photoexcited dimers and RET among aggregates would be of paramount importance in the development of new functional materials.^73^

## Data Availability

The data that support the findings of this study are available from the corresponding author upon reasonable request.

## Supporting Information Available

Displaced bosonic creation and annihilation operators; rotation of dimer electronic Hamiltonian on the adiabatic mean field basis; complete expressions of Redfield tensor terms; additional results obtained for Debye spectral density, for centrosymmetric dimers, and for monochromatic initial excitation; comparison with spectra obtained through sum over state approach; population fitting for rates extrapolation; *P*_*M*_1_^*^_ operator definition (PDF)
