# Supplementary material for: A Comprehensive Approach to Exciton Delocalization and Energy Transfer
Source: J Chem Theory Comput. 2022 Dec 23;19(2):436–47. doi: 10.1021/acs.jctc.2c00980 (PMC9878730; doi:10.1021/acs.jctc.2c00980)
Supplement: Supplementary file 1 — ct2c00980_si_001.pdf [file ct2c00980_si_001.pdf]

Supplementary Information

A comprehensive approach to exciton delocalization and energy transfer

Davide Giavazzi, Sangeeth Saseendran, Francesco Di Maiolo,* and Anna Painelli

Department of Chemistry, Life Science and Environmental Sustainability, Università di Parma, 43124 Parma, Italy

E-mail: francesco.dimaiolo@unipr.it

S1 Displaced bosonic creation and annihilation operators

As stated in Sec. 2 (main text), the equilibrium position for each harmonic oscillator varies with the ionicity on the relevant molecule:

$$\bar{Q}_i = \langle G_i|\hat{Q}_i|G_i\rangle = \sqrt{\frac{2\omega_{v,i}}{\hbar}}\frac{g_i}{\omega_{v,i}^2}\bar{\rho}_i \tag{1}$$

It is convenient to move the origin of the vibrational coordinate to the equilibrium position, via a Lang-Firsov transformation of the vibrational operators:[1,2]

$$\hat{\tilde{Q}}_i = \hat{Q}_i - \bar{Q}_i = \sqrt{\frac{\hbar}{2\omega_v}}\left(\hat{\tilde{a}}_i^\dagger + \hat{\tilde{a}}_i\right) \tag{2}$$

$$\hat{\tilde{P}}_i = \hat{P}_i = i\sqrt{\frac{\hbar\omega_v}{2}}\left(\hat{\tilde{a}}_i^\dagger - \hat{\tilde{a}}_i\right) \tag{3}$$

where we introduced displaced bosonic creation and annihilation operators, $\hat{\tilde{a}}_i$ and $\hat{\tilde{a}}_i^\dagger$.

S2 Rotation of the dimer electronic Hamiltonian on the adiabatic mean field basis

The electronic part of Hamiltonian in Eq. 4 (main text) that describes a pair of dipolar dyes on the diabatic $|N_1, N_2\rangle$, $|N_1, Z_2\rangle$, $|Z_1, N_2\rangle$ and $|Z_1, Z_2\rangle$ basis can be rewritten as follows:

$$
\begin{aligned}
\hat{H} &= 2z_1\hat{\rho}_1 - \tau_1\hat{\sigma}_{x,1} + 2z_2\hat{\rho}_2 - \tau_2\hat{\sigma}_{x,2} + V\hat{\rho}_1\hat{\rho}_2 \\
&= 2z_1\left(\frac{1}{2} - \frac{\hat{\sigma}_{z,1}}{2}\right) - \tau_1\hat{\sigma}_{x,1} + 2z_2\left(\frac{1}{2} - \frac{\hat{\sigma}_{z,2}}{2}\right) - \tau_2\hat{\sigma}_{x,2} \\
&+ V\left(\frac{1}{2} - \frac{\hat{\sigma}_{z,1}}{2}\right)\left(\frac{1}{2} - \frac{\hat{\sigma}_{z,2}}{2}\right)
\end{aligned}
\tag{4}
$$

where, for each molecule, we define the operators:

$$
\hat{\sigma}_x = \begin{pmatrix} 0 & 1 \\ 1 & 0 \end{pmatrix}, \quad \hat{\sigma}_z = \begin{pmatrix} 1 & 0 \\ 0 & -1 \end{pmatrix}, \quad \hat{\rho} = \begin{pmatrix} 0 & 0 \\ 0 & 1 \end{pmatrix} = \left(\frac{1}{2} - \frac{\hat{\sigma}_z}{2}\right) \tag{5}
$$

For each molecule, we define two adiabatic states $|G_i\rangle$ and $|E_i\rangle$, linear combination of the $|N_i\rangle$ and $|Z_i\rangle$:

$$
\begin{aligned}
|G_i\rangle &= \sqrt{1-\bar{\rho}_i}|N_i\rangle + \sqrt{\bar{\rho}_i}|Z_i\rangle \\
|E_i\rangle &= \sqrt{\bar{\rho}_i}|N_i\rangle - \sqrt{1-\bar{\rho}_i}|Z_i\rangle.
\end{aligned}
$$

To rotate the Hamiltonian in Eq. 4 on the adiabatic basis for the dimer ($|G_1, G_2\rangle$, $|G_1, E_2\rangle$, $|E_1, G_2\rangle$ and $|E_1, E_2\rangle$), we introduce the following Paulion operators, $\hat{p}_i^{(\dagger)}$ that transform the i-th site from $|E_i\rangle$ to $|G_i\rangle$ (from $|G_i\rangle$ to $|E_i\rangle$). These operators are related to the operators in Eq. 5 as follows:[2,3]

$$
\hat{\sigma}_{z,i} = (1-2\bar{\rho}_i)\left(1 - 2\hat{p}_i^\dagger\hat{p}_i\right) - 2\sqrt{\bar{\rho}_i(1-\bar{\rho}_i)}\left(\hat{p}_i^\dagger + \hat{p}_i\right) \tag{6}
$$

$$
\hat{\sigma}_{x,i} = 2\sqrt{\bar{\rho}_i(1-\bar{\rho}_i)}\left(1 - 2\hat{p}_i^\dagger\hat{p}_i\right) + (1-2\bar{\rho}_i)\left(\hat{p}_i^\dagger + \hat{p}_i\right) \tag{7}
$$

By plugging Eqs. 6 and 7 into Eq. 4 and neglecting constant terms, we get:

$$\hat{H} = \hat{H}_{mf} + \hat{H}_{exc} + \hat{H}_{uex} \tag{8}$$

$$\begin{aligned}\hat{H}_{mf} = &\left[2(1-2\bar{\rho}_1)(z_1+M\bar{\rho}_2)+4\tau_1\sqrt{\bar{\rho}_1(1-\bar{\rho}_1)}\right]\hat{n}_1 \\ &+\left[2(1-2\bar{\rho}_2)(z_2+M\bar{\rho}_1)+4\tau_2\sqrt{\bar{\rho}_2(1-\bar{\rho}_2)}\right]\hat{n}_2 \\ &+\left[2\sqrt{\bar{\rho}_1(1-\bar{\rho}_1)}(z_1+M\bar{\rho}_2)-\tau_1(1-2\bar{\rho}_1)\right]\left(\hat{p}_1^\dagger+\hat{p}_1\right) \\ &+\left[2\sqrt{\bar{\rho}_2(1-\bar{\rho}_2)}(z_2+M\bar{\rho}_1)-\tau_2(1-2\bar{\rho}_2)\right]\left(\hat{p}_2^\dagger+\hat{p}_2\right)\end{aligned} \tag{9}$$

$$\begin{aligned}\hat{H}_{exc} =&V\sqrt{\bar{\rho}_1(1-\bar{\rho}_1)}\sqrt{\bar{\rho}_2(1-\bar{\rho}_2)}\left(\hat{p}_1^\dagger\hat{p}_2+\hat{p}_1\hat{p}_2^\dagger\right) \\ &+V(1-2\bar{\rho}_1)(1-2\bar{\rho}_2)\hat{n}_1\hat{n}_2\end{aligned} \tag{10}$$

$$\begin{aligned}\hat{H}_{uex} =&V\sqrt{\bar{\rho}_1(1-\bar{\rho}_1)}\sqrt{\bar{\rho}_2(1-\bar{\rho}_2)}\left(\hat{p}_1^\dagger\hat{p}_2^\dagger+\hat{p}_1\hat{p}_2\right) \\ &+V(1-2\bar{\rho}_1)\sqrt{\bar{\rho}_2(1-\bar{\rho}_2)}\left(\hat{p}_2^\dagger+\hat{p}_2\right)\hat{n}_1 \\ &+V(1-2\bar{\rho}_2)\sqrt{\bar{\rho}_1(1-\bar{\rho}_1)}\left(\hat{p}_1^\dagger+\hat{p}_1\right)\hat{n}_2\end{aligned} \tag{11}$$

where $\hat{n}_i = \hat{p}_i^\dagger\hat{p}_i$ and $M = V/2$.

This expression holds true for any choice of $\bar{\rho}_1$ and $\bar{\rho}_2$ value. In order to find the $|G_i\rangle$ and $|E_i\rangle$ basis states that describe the actual ground and excited states of the two molecules in the environment of the dimer (i.e., feeling the potential generated by the other molecule), we set to zero the last two terms in Eq. 9:

$$\begin{cases} 2\sqrt{\bar{\rho}_1(1-\bar{\rho}_1)}(z_1+M\bar{\rho}_2)-\tau_1(1-2\bar{\rho}_1)=0 \\ 2\sqrt{\bar{\rho}_2(1-\bar{\rho}_2)}(z_2+M\bar{\rho}_1)-\tau_2(1-2\bar{\rho}_2)=0 \end{cases} \tag{12}$$

thus getting:

$$\begin{cases} \bar{\rho}_2 = \frac{\tau_1(1-2\bar{\rho}_1)}{2M\sqrt{\bar{\rho}_1(1-\bar{\rho}_1)}} - z_1/M \\ \bar{\rho}_1 = \frac{\tau_2(1-2\bar{\rho}_2)}{2M\sqrt{\bar{\rho}_2(1-\bar{\rho}_2)}} - z_2/M \end{cases} \tag{13}$$

Eq. 9 finally reduces to:

$$\hat{H}_{mf} = \left[\frac{\tau_1}{\sqrt{\bar{\rho}_1(1-\bar{\rho}_1)}}\right]\hat{n}_1 + \left[\frac{\tau_2}{\sqrt{\bar{\rho}_2(1-\bar{\rho}_2)}}\right]\hat{n}_2 \tag{14}$$

which is the Hamiltonian of two non interacting molecules, each one feeling the electric field generated by the other one. Eq. 14 together with Eqs. 10 and 11, give Eq. 8 of the main text, with the mean field transition energies of the dyes reading:

$$\Delta\mathcal{E}_{CT,i} = \frac{\tau_i}{\sqrt{\bar{\rho}_i(1-\bar{\rho}_i)}} \tag{15}$$

S3 Redfield relaxation tensor

For a given bath and system-bath interaction hamiltonian $\hat{H}_B$ and $\hat{H}_{SB}$ ($\hat{H}_{bath}$ and $\hat{H}_{dimer-bath}$ in the main text), the terms of the four-dimensional Redfield relaxation tensor $R_{ab,cd}$ read:

$$R_{ab,cd} = -\delta_{d,b}\sum_e \Gamma^+_{ae,ec} - \delta_{a,c}\sum_e \Gamma^-_{de,eb} + \Gamma^+_{db,ac} + \Gamma^-_{db,ac} \tag{16}$$

with the kinetic coefficients reading:

$$\begin{aligned}
\Gamma^+_{db,ac} &= \frac{1}{\hbar^2}\int_0^\infty d\tau e^{-i\omega_{ac}\tau}\langle(\hat{H}_{SB}(0))_{db}\hat{H}_{SB}(-\tau))_{ac}\rangle_{bath} \\
\Gamma^-_{db,ac} &= \frac{1}{\hbar^2}\int_0^\infty d\tau e^{-i\omega_{db}\tau}\langle(\hat{H}_{SB}(-\tau))_{db}\hat{H}_{SB}(0))_{ac}\rangle_{bath}
\end{aligned} \tag{17}$$

where the angle brackets $\langle\cdot\rangle_{bath}$ indicate the average over the equilibrium bath states and $\hat{H}_{SB}(\tau) = e^{i\hat{H}_B\tau}\hat{H}_{SB}e^{-i\hat{H}_B\tau}$. For the $\hat{H}_{bath}$ and $\hat{H}_{dimer-bath}$ defined in the main text, $\Gamma^+_{db,ac}$ and $\Gamma^-_{db,ac}$ read:

$$\begin{aligned}
\Gamma^+_{db,ac} =& \frac{\pi}{\hbar^2}q^{1/+}_{db}q^{1/+}_{ac}\left[\mathcal{I}_{1/+}(\omega_{ac})\langle\hat{n}(\omega_{ac})\rangle_b + \mathcal{I}_{1/+}(\omega_{ca})\langle\hat{n}(\omega_{ca})+1\rangle_b\right] \\
&+ \frac{\pi}{\hbar^2}q^{2/-}_{db}q^{2/-}_{ac}\left[\mathcal{I}_{2/-}(\omega_{ac})\langle\hat{n}(\omega_{ac})\rangle_b + \mathcal{I}_{2/-}(\omega_{ca})\langle\hat{n}(\omega_{ca})+1\rangle_b\right]
\end{aligned} \tag{18}$$

$$\Gamma^{-}_{db,ac} = \frac{\pi}{\hbar^2} q^{1/+}_{db} q^{1/+}_{ac} \left[\mathcal{I}_{1/+}(\omega_{bd})\langle\hat{n}(\omega_{bd})\rangle_b + \mathcal{I}_{1/+}(\omega_{db})\langle\hat{n}(\omega_{db})+1\rangle_b\right] + \frac{\pi}{\hbar^2} q^{2/-}_{db} q^{2/-}_{ac} \left[\mathcal{I}_{2/-}(\omega_{bd})\langle\hat{n}(\omega_{bd})\rangle_b + \mathcal{I}_{2/-}(\omega_{db})\langle\hat{n}(\omega_{db})+1\rangle_b\right] \tag{19}$$

where $q^{1/+(2/-)}_{db}$ and $q^{1/+(2/-)}_{ac}$ are the db and ac matrix elements of the vibrational operators $\hat{Q}_{1/+(2/-)}$, two spectral densities are defined as $\mathcal{I}_{1/+}(\omega) = \sum_i |g_i|^2 \delta(\omega - \omega_i)$ and $\mathcal{I}_{2/-}(\omega) = \sum_j |f_j|^2 \delta(\omega - \omega_j)$ and $\langle\hat{n}(\omega_{ac})\rangle_b = \left(e^{\frac{\hbar\omega_{ac}}{kT}} - 1\right)^{-1}$ is the Bose-Einstein distribution function, where we considered $\langle\hat{n}(\omega_{ac})\rangle_{b1/+} = \langle\hat{n}(\omega_{ac})\rangle_{b2/-} = \langle\hat{n}(\omega_{ac})\rangle_b$.

Since the integrals in Eq. 17 span positive frequencies, Eqs. 18 and 19 have to be read as:

$$\Gamma^{+}_{db,ac} = \frac{\pi}{\hbar^2}\left[q^{1/+}_{db} q^{1/+}_{ac} \mathcal{I}_{1/+}(\omega_{ac})\langle\hat{n}(\omega_{ac})\rangle_b + q^{2/-}_{db} q^{2/-}_{ac} \mathcal{I}_{2/-}(\omega_{ac})\langle\hat{n}(\omega_{ac})\rangle_b\right] \quad \text{for} \quad \varepsilon_a > \varepsilon_c$$

$$\Gamma^{+}_{db,ac} = \frac{\pi}{\hbar^2}\left[q^{1/+}_{db} q^{1/+}_{ac} \mathcal{I}_{1/+}(\omega_{ca})\langle\hat{n}(\omega_{ca})+1\rangle_b + q^{2/-}_{db} q^{2/-}_{ac} \mathcal{I}_{2/-}(\omega_{ca})\langle\hat{n}(\omega_{ca})+1\rangle_b\right] \quad \text{for} \quad \varepsilon_a < \varepsilon_c \tag{20}$$

$$\Gamma^{-}_{db,ac} = \frac{\pi}{\hbar^2}\left[q^{1/+}_{db} q^{1/+}_{ac} \mathcal{I}_{1/+}(\omega_{bd})\langle\hat{n}(\omega_{bd})\rangle_b + q^{2/-}_{db} q^{2/-}_{ac} \mathcal{I}_{2/-}(\omega_{bd})\langle\hat{n}(\omega_{bd})\rangle_b\right] \quad \text{for} \quad \varepsilon_b > \varepsilon_d$$

$$\Gamma^{-}_{db,ac} = \frac{\pi}{\hbar^2}\left[q^{1/+}_{db} q^{1/+}_{ac} \mathcal{I}_{1/+}(\omega_{db})\langle\hat{n}(\omega_{db})+1\rangle_b + q^{2/-}_{db} q^{2/-}_{ac} \mathcal{I}_{2/-}(\omega_{db})\langle\hat{n}(\omega_{db})+1\rangle_b\right] \quad \text{for} \quad \varepsilon_b < \varepsilon_d \tag{21}$$

while for $\varepsilon_a = \varepsilon_c$ and $\varepsilon_b = \varepsilon_d$ the Redfield kinetic coefficients vanish as we impose $\mathcal{I}_{1/2/+/-}(\omega = 0) = 0$.

S4 Additional results

S4.1 Debye spectral density

We consider the Debye spectral density:

$$\mathcal{I}(\omega) = \frac{\hbar^2}{\pi} \frac{\omega/\omega_c}{1+\omega^2/\omega_c^2}\eta \tag{22}$$

where η measures the strength of the system-bath coupling and ω_c is the cut-off frequency.

For homodimers, the η parameter is adjusted as to have at ω_+ the same value of the

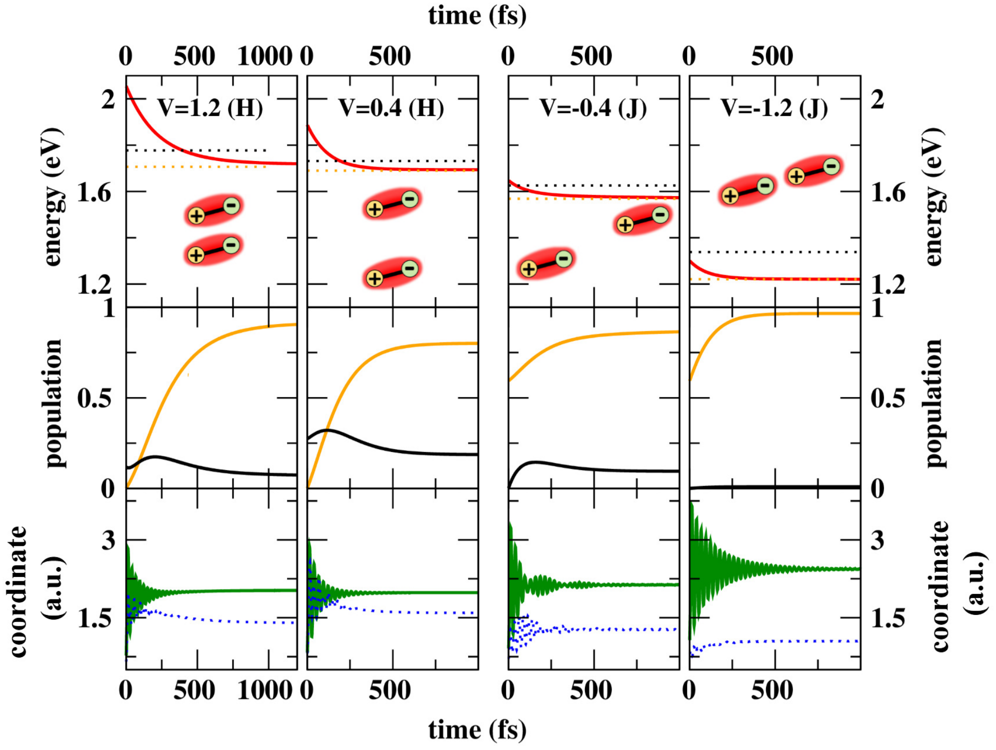

Figure 1S: Results for Nile Red dimers $(NR)_2$ (molecular parameters in Tab. 1, main text) obtained for different V values (left panels: H-dimers; right panels: J-dimers), accounting for a Debye bath spectral density. Top panels: time evolution of the system energy (red). For reference, the energy of the lowest vibronic eigenstates in S_1 and S_2 manifolds are shown as orange and black dotted lines, respectively. Middle panels: time evolution of the populations of the lowest vibronic eigenstate in S_1 and S_2 manifolds (orange and black lines, respectively). Bottom panels: time evolution of $\langle \hat{Q}_+ \rangle$, and of ΔQ_- (green and blue dotted lines, respectively).

constant spectral density used in the main text ($\eta = \gamma\frac{1+\omega_+^2/\omega_c^2}{\omega_+/\omega_c}$ with $\gamma = 5$ ps^{-1}) and $\omega_c = 0.5$ eV. Relevant results for a Nile Red dimer $(NR)_2$ for different values of the intermolecular interaction strength V are shown in Fig. 1S.

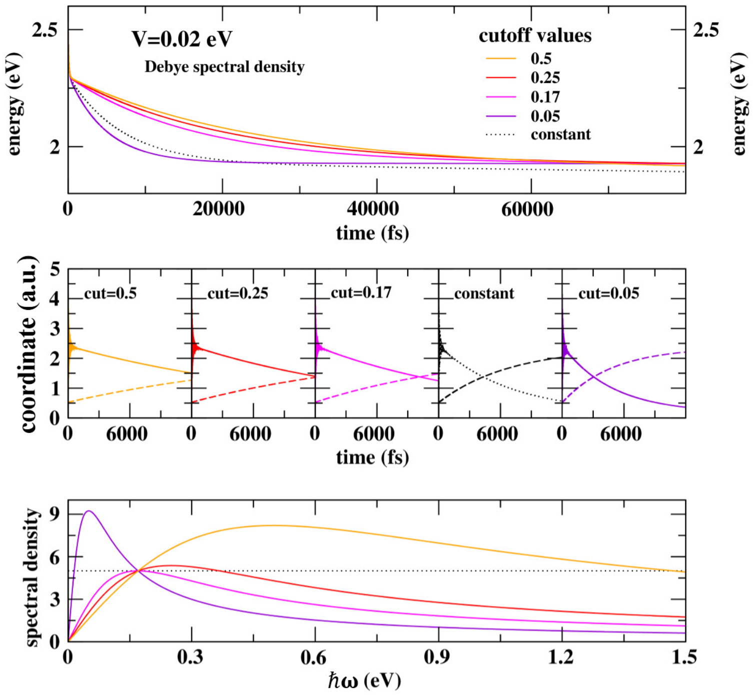

Figure 2S: RET dynamics for a DANS-NR pair (molecular parameters in Tab. 1, main text) calculated for V=0.02 eV with a Debye spectral density for different cut-off frequencies. Top panel: energy of the system as a function of time; middle panels: relevant time evolution of $\langle \hat{Q}_{\mathcal{D}} \rangle$ (continuous lines) and $\langle \hat{Q}_{\mathcal{A}} \rangle$ (dashed lines); bottom panel: different bath spectral densities used (in unit of $\hbar/\pi$; the black dotted line refers to the constant spectral density used in the main text).

Figure 2S shows results for a DANS-NR RET pair with V=0.02 eV and a Debye spectral density for different ω_c values. The η parameter is adjusted as to always have the same value of the spectral density at ω_c. The same spectral density is used for both donor and acceptor. RET velocity depends on the values of the spectral density at frequencies lower than the energy donor vibrational frequency.

S4.2 Centrosymmetric dimers

We always consider systems of aligned molecules, where a positive electrostatic interaction sets the in-phase combination $|G_1E_2\rangle + |E_1G_2\rangle$ at higher energy than the out-of-phase one $|G_1E_2\rangle - |E_1G_2\rangle$, and *vice versa* for a negative interaction. As discussed in the main text, for two molecules oriented in the same direction (non-centrosymmetric dimers), the bright state reached upon light absorption is the in-phase combination (left part of Fig. 3S reports for reference results also shown in Fig. 2 of the mian text). On the other hand, for molecules oriented in opposite directions, the out-of-phase combination becomes the bright state. The rightmost part of figure 3S shows results for centrosymmetric dimers. More specifically, the systems in the first and third column have the same Hamiltonian (positive electrostatic interactions), but the coherent excitation populates S_2 in the non-centrosymmetric system (first column) and S_1 in the centrosymmetric one (third column), as it can be seen from the populations reported in the middle panels. Moreover, the expectation value of $\hat{Q}_+$ shows how in the centrosymmetric dimer the coherence is maintained for a longer time respect to the non-censtrosymmetric system, since all the excited state dynamics occurs in the same electronic manifold. The same considerations apply to the systems with negative interactions (second and fourth column). Here the slower relaxation of the centrosymmetric system is due to the higher energy difference between S_1 and S_2.

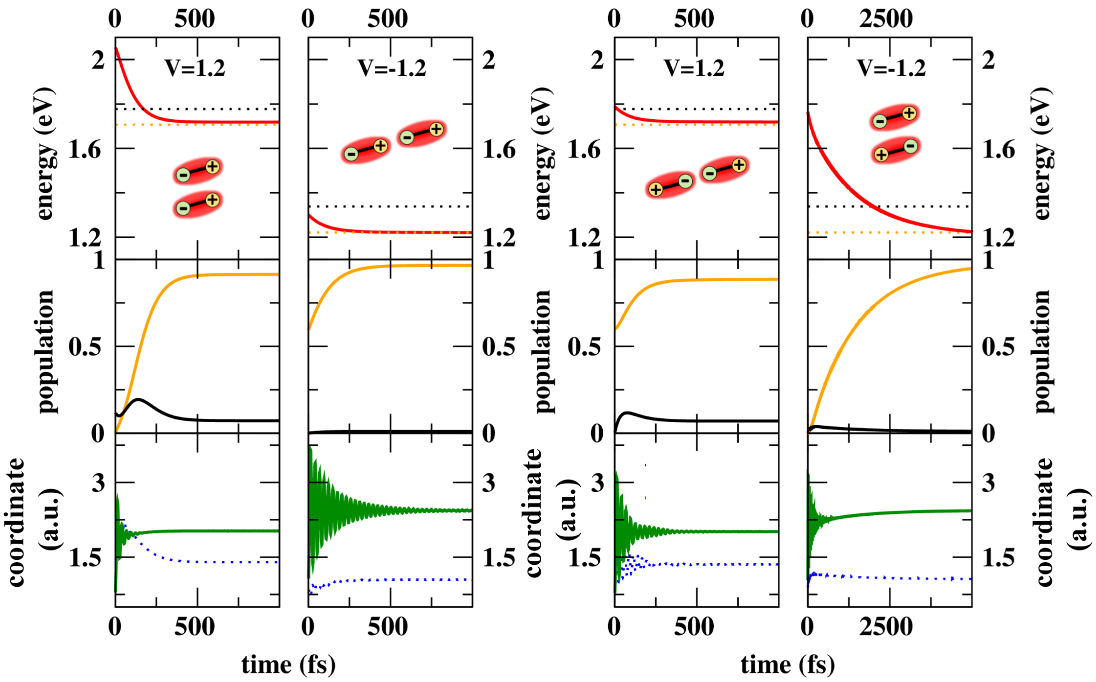

Figure 3S: Results for dimers of NR (molecular parameters in Tab. 1, main text) with different relative orientations of the monomers (sketches of the dimeric geometries are shown in the top panels; left panels are taken from Fig. 2 in the main text). Top panels: time evolution of the system energy (red). For reference, the energy of the lowest vibronic eigenstates in S_1 and S_2 manifolds are shown as orange and black dotted lines, respectively. Middle panels: time evolution of the populations of the lowest vibronic eigenstate in S_1 and S_2 manifolds (orange and black lines, respectively). Bottom panels: time evolution of $\langle \hat{Q}_+ \rangle$, and of ΔQ_- (green and blue dotted lines, respectively).

S4.3 Steady state optical spectra

We compare the absorption and long time (i.e., after 1 ps) emission spectra obtained from the Liouville-von Neumann dynamics and the steady state spectra calculated through a sum-over-states (SOS) approach. In particular, the Hamiltonian in Eq. 4 in the main text is diagonalized (molecular parameters for Nile Red dimers are reported in the main text) and the absorption spectra are calculated according to:

$$A(\hbar\omega) \propto \hbar\omega \sum_{i>1} |\mu_{i1}|^2 \frac{\sigma}{4\left(\hbar\omega - \hbar\omega_{i1}\right)^2 + \sigma^2} \tag{23}$$

where we assumed that only the lowest vibronic eigenstate is populated at ambient temperature, i runs over the vibronic eigenstates and a lorentzian bandshape with standard deviation σ is associated with every transition. Similarly, once the Kasha's state $|f\rangle$ is identified, steady state emission spectra are calculated as:

$$F(\hbar\omega) \propto (\hbar\omega)^3 \sum_{i\geq f} \sum_{a<i} |\mu_{ai}|^2 \frac{\sigma}{4\left(\hbar\omega - \hbar\omega_{ai}\right)^2 + \sigma^2} \exp\left(\frac{\hbar\omega_{if}}{k_b T}\right) \tag{24}$$

where we introduced the Boltzmann distribution to account for the possible thermal population of the excited vibronic eigenstates. Figure 4S shows how the absorption and emission (collected after 1 ps) spectra calculated with the Liouville-von Neumann equation nicely match those obtained using Eqs. 23 and 24.

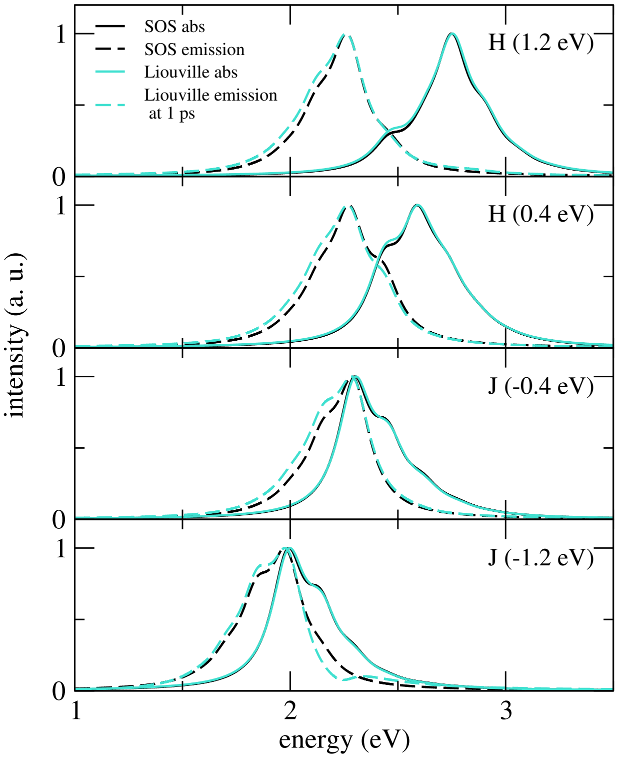

Figure 4S: Nile Red dimers spectra for different V values (eV units). Each panel compares the spectra calculated in the main text (cyan curves) with those calculated using Eqs. 23 and 24 (black curves). Continuous curves refer to absorption spectra, whereas dashed ones refer to fluorescence spectra. All model parameters are the same as in the main text. In Eqs. 23 and 24 we set $\sigma = 0.8$ eV and $T = 298$ K.

S4.4 Monochromatic excitation

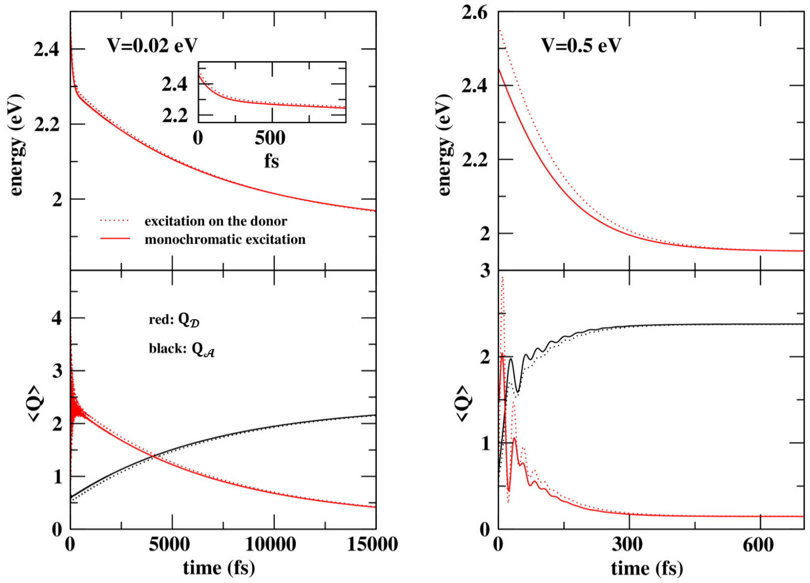

Figure 5S: Comparison between selective coherent excitation of the energy donor (using only $\hat{\mu}_{\mathcal{D}}$; dotted lines) and a monochromatic excitation simulated with a gaussian centered at 2.9 eV with HWHM = 0.1 eV (using $\hat{\mu}_{\mathcal{D}} + \hat{\mu}_{\mathcal{A}}$; continuous lines) for a DANS-Nile Red pair (molecular parameters reported in the main text) for V=0.02 eV (left panels) and V=0.5 eV (right panels). Top panels: Energy of the system as a function of time; bottom panels: Time evolution of $\langle\hat{Q}_{\mathcal{D}}\rangle$ (red lines) and $\langle\hat{Q}_{\mathcal{A}}\rangle$ (black lines).

S5 Population fitting for rates extrapolation

Energy transfer rates from the dynamical simulations are obtained fitting the time evolution of the $\mathcal{DA}^*$ population (eigenstate 106 in all cases) with the equation:

$$f(t) = a_1 - a_2 \exp(-a_3 t) \tag{25}$$

where a_3 is the rate of interest.

Fitting parameters relevant to the rates shown in Figs. 6 and 8 of the main text are reported in Tables 1 and 2, respectively.

Table 1: Fitting parameters for the extraction of the RET rates shown in Fig. 6 of the main text.

V [eV]	a_1	a_2	a_3 [ps^{-1}]
0.005	0.906701	0.908892	$1.15146 \cdot 10^{-2}$
0.01	0.951624	0.95972	$4.27418 \cdot 10^{-2}$
0.02	0.985505	1.0112	0.14544
0.03	0.99293	1.03814	0.27217
0.05	0.99553	1.07835	0.57373
0.08	0.996217	1.12626	1.07081
0.1	0.99942	1.15303	1.378
0.2	1.01033	1.2237	2.63451
0.3	1.01176	1.2501	3.57676
0.5	1.00748	1.25403	4.62856
0.8	1.00496	1.24491	4.94457
1.0	1.00415	1.24407	4.98588

Table 2: Fitting parameters for the extraction of the RET rates shown in Fig. 8 of the main text.

z_D [eV]	a_1	a_2	a_3 [ps^{-1}]
1.250	0.980864	0.978786	0.0788802
1.270	0.983235	0.970831	0.74767
1.282	0.994785	1.15877	2.40726
1.291	0.998026	1.22714	4.28024
1.303	0.99831	1.22271	4.17772
1.315	0.994807	1.06028	0.443007
1.320	0.99293	1.03814	0.27217
1.326	0.989916	1.01532	0.15287
1.332	0.985235	0.992547	0.10539
1.338	0.987335	1.00007	0.0850167
1.344	0.977574	0.967978	0.0828968
1.356	0.975398	0.927693	0.208301
1.359	0.98663	1.02028	0.63985
1.373	0.999291	1.25588	3.82228
1.385	0.999392	1.26041	4.00859
1.396	0.996697	1.21371	3.20617
1.409	0.995902	1.08252	0.57377
1.419	0.990961	1.02157	0.16974

S6 $\hat{P}_{M_1^*}$ operator definition

In Sec. 4.3 in the main text, the total amount of electronic excitation over one of the two molecules of the dimer is evaluated by the following operator:

$$\hat{P}_{M_1^*} = \rho_1 |N_1\rangle\langle N_1| + (1-\rho_1)|Z_1\rangle\langle Z_1| - \sqrt{\rho_1(1-\rho_1)}(|N_1\rangle\langle Z_1| + |Z_1\rangle\langle N_1|) \tag{26}$$

where $\rho_1 = \langle\phi_1|Z_1\rangle\langle Z_1|\phi_1\rangle$, $|\phi_1\rangle$ being the ground state of the dimer Hamiltonian (Eq. 4 in the main text).

References

(1) Feinberg, D.; Ciuchi, S.; de Pasquale, F. Squeezing phenomena in interacting electron-phonon systems. *International Journal of Modern Physics B* **1990**, *04*, 1317–1367.

(2) Anzola, M.; Painelli, A. Aggregates of polar dyes: beyond the exciton model. *Physical Chemistry Chemical Physics* **2021**, *23*, 8282–8291.

(3) Terenziani, F.; Painelli, A. Time-resolved spectra of polar–polarizable chromophores in solution. *Chemical Physics* **2003**, *295*, 35–46.
